# A Novel Weighted Support Vector Machine Based on Particle Swarm Optimization for Gene Selection and Tumor Classification

**DOI:** 10.1155/2012/320698

**Published:** 2012-07-26

**Authors:** Mohammad Javad Abdi, Seyed Mohammad Hosseini, Mansoor Rezghi

**Affiliations:** ^1^Department of Computer Sciences, Faculty of Mathematical Sciences, Tarbiat Modares University, P.O. Box 14115-134, Tehran, Iran; ^2^Department of Applied Mathematics, Sahand University of Technology, Tabriz, Iran

## Abstract

We develop a detection model based on support vector machines (SVMs) and particle swarm optimization (PSO) for gene selection and tumor classification problems. The proposed model consists of two stages: first, the well-known minimum redundancy-maximum relevance (mRMR) method is applied to preselect genes that have the highest relevance with the target class and are maximally dissimilar to each other. Then, PSO is proposed to form a novel weighted SVM (WSVM) to classify samples. In this WSVM, PSO not only discards redundant genes, but also especially takes into account the degree of importance of each gene and assigns diverse weights to the different genes. We also use PSO to find appropriate kernel parameters since the choice of gene weights influences the optimal kernel parameters and vice versa. Experimental results show that the proposed mRMR-PSO-WSVM model achieves highest classification accuracy on two popular leukemia and colon gene expression datasets obtained from DNA microarrays. Therefore, we can conclude that our proposed method is very promising compared to the previously reported results.

## 1. Introduction

Microarray technology is a tool for analyzing gene expressions consisting of a small membrane containing samples of many genes arranged in a regular pattern. Microarrays may be used to assay gene expression within a single sample or to compare gene expression in two different cell types or tissue samples, such as in healthy and cancerous tissue. The use of this technology is increased in recent years to identify genes involved in the development of diseases. Various clustering, classification, and prediction techniques have been utilized to analyze, classify, and understand the gene expression data such as Fisher discriminant analysis [1], artificial neural networks [2], and support vector machines (SVM) [3]. Briefly, SVM is a supervised learning algorithm based on statistical learning theory introduced by Vapnik [4]. It has great performance since it can handle a nonlinear classification efficiently by mapping samples from low dimensional input space into high dimensional feature space with a nonlinear kernel function. It is useful in handling classification tasks for high-dimensional and sparse microarray data and has been recommended as an effective approach to treat this specific data structure [5–8]. Due to its many attractive characters, it has been also widely used in various fields such as image recognition, text classification, speaker identification, and medical diagnosis, bioinformatics. Therefore, our study intends to investigate the application of SVM in tumor classification problem and suggests an effective model to minimize its error rate.

It is well known that SVM assumed that all the available genes of certain gene expression data have equal weights in classification process. However, for a real tumor classification problem each gene may possess different relevance to the classification results. Thus, the genes with more relevance are more important than those with less relevance. Usually, there are two approaches to tackle this issue. One strategy is gene selection aiming at determination of a subset of genes which is most discriminative and informative for classification. The other is gene weighting which seeks to estimate the relative importance of each gene and assign it a corresponding weight [9–11]. Gene selection has attracted increasing interests in bioinformatics in recent years because its results can effectively help cancer diagnosis and clinical treatment. In this case, many outstanding methods based on particle swarm optimization (PSO) have been developed. PSO is a new evolutionary computation technique proposed by Kennedy and Eberhart [12] which was motivated by simulations of bird flocking or fish schooling. Shen et al. [8] introduced a combination of PSO and support vector machines (SVMs) for gene selection and tumor classification problem. In their work, the modified discrete PSO was applied to select genes and SVM to diagnose colon tumor. They also proposed a combination of PSO and tabu search (TS) approaches for gene selection problem [13]. The combination of TS as a local improvement procedure and PSO enabled their algorithm to overleap local optima and showed satisfactory performance. In 2008, Chuang et al. [14] suggested an improved binary PSO. The main contribution of their work was resetting all the global best particle positions after no change in three consecutive iterations. Li et al. [15] introduced a novel hybrid of PSO and genetic algorithms (GA) for the same purpose, overcoming the local optimum problem.

On the other hand, instead of making a binary decision on a genes' relevance, gene weighting utilizes a continuous value and hence has a finer granularity in determining the relevance. The strategy proposed in this work is a combination of gene selection and gene weighting. The proposed method consists of two stages. First, we apply minimum redundancy-maximum relevance (mRMR) method, proposed by Hanchuan et al. [16], to preselect genes having the highest relevance with the target class and being maximally dissimilar to each other. Then, PSO is employed to form a novel weighted SVM (WSVM) to classify samples. In this WSVM, PSO not only discards redundant genes (gene selection), but also especially takes into account the degree of importance of each gene and assigns diverse weights to the different genes (gene weighting). To construct an accurate SVM, we also use PSO to find appropriate kernel parameters, since the choice of gene weights influences the optimal kernel parameters and vice versa. Experimental results show that our proposed method (called mRMR-PSO-WSVM) achieves higher classification rate than previously reported results.

The rest of this paper is organized as follows. The following section provides a brief description of the well-known mRMR filter method, SVM classifier, weighted SVM and PSO besides the proposed method, respectively. Experimental results and conclusions are demonstrated in Sections 3 and 4, respectively.

## 2. Method

### 2.1. Minimum Redundancy-Maximum Relevance (mRMR)

In this work a well-designed filter method, mRMR, is employed to enhance the gene selection in achieving both high accuracy and fast speed. In high-dimensional microarray data, due to the existence of a set of several thousands of genes, it is hard and even infeasible for SVM to be trained accurately. Alternative methods should be effectively applied to tackle this problem. Therefore, first of all, mRMR is applied to filter noisy and redundant genes. More specifically, mRMR method [16] is a criterion for first-order incremental gene selection, which is warmly being studied by a great number of researchers. In mRMR, genes which have both minimum redundancy for input genes and maximum relevancy for disease classes should be selected. Thus this method is based on two important metrics. One is mutual information between disease classes and each gene, which is used to measure the relevancy, and the other is mutual information between every two genes, which is employed to compute the redundancy. Let *S* denote the subset of selected genes, and *Ω* is the set of all available genes; the minimum redundancy can be computed by

(1) $$\begin{array}{c} \underset{S\subset \Omega }{\min}\frac{1}{{\left|S\right|}^{2}}\sum_{i,j\in S}I\left(g_{i},g_{j}\right), \end{array}$$

where *I*(*g*_*i*_, *g*_*j*_) is the mutual information between *i*th and *j*th genes which measures the mutual dependence of these two variables. Formally, the mutual information of two discrete random variables *g*_*i*_ and *g*_*j*_ can be defined as

(2) $$\begin{array}{c} I\left(g_{i},g_{j}\right)=\sum_{m\in g_{i}}\;\sum_{n\in g_{j}}p\left(m,n\right)\log\frac{p\left(m,n\right)}{p\left(m\right)p\left(n\right)}, \end{array}$$

where *p*(*m*, *n*) is the joint probability distribution function of *g*_*i*_ and *g*_*j*_, and *p*(*m*) and *p*(*n*) are the marginal probability distribution functions of *g*_*i*_ and *g*_*j*_, respectively [17]. In (4), |*S*| is the number of genes of *S*. In contrast, mutual information *I*(*T*, *g*_*j*_) is usually employed to calculate discrimination ability from gene *g*_*i*_ to class *T* = {*t*_1_, *t*_2_}, where *t*_1_ and *t*_2_ denote the healthy and tumor classes. Therefore, the maximum relevancy can be calculated by

(3) $$\begin{array}{c} \underset{S\subset \Omega }{\max}\frac{1}{\left|S\right|}\sum_{i\in S}I\left(T,g_{i}\right). \end{array}$$

Combined (5) with (6), mRMR feature selection criterion can be obtained as below in difference form:

(4) $$\begin{array}{c} \underset{S\subset \Omega }{\max}\left\{\sum_{i\in S}I\left(T,g_{i}\right)-\left[\frac{1}{\left|S\right|}\sum_{i,j\in S}I\left(g_{i},g_{j}\right)\right]\right\}. \end{array}$$

### 2.2. Support Vector Machines (SVM)

SVM classifier is briefly described as follows [18, 19]. Assume {*x*_*i*_, *y*_*i*_}_*i*=1_^*N*^ is a training dataset, where *x* is the input sample, and *y* ∈ {+1, −1} is the label of classes. The SVM aim is to determine a hyper plane that optimally separates two classes using training dataset. This hyper plane is defined as *w* · *x* + *b* = 0, where *x* is a point lying on the hyper plane, *w* determines the orientation of the hyper plane, and *b* is the bias of the distance of hyper plane from the origin. To find the optimum hyper plane, ||*w*||^2^ must be minimized under the constraint *y*_*i*_(*w* · *x*_*i*_ + *b*) ≥ 1, *i* = 1,2,…, *n*. Therefore, it is required to solve the optimization problem given by

(5) min⁡ 12||w||2s.t. yi(w·xi+b)≥1, i=1,2,…,n.

Now, the positive slack variables *ξ*_*i*_ are introduced to substitute in the optimization problem and allow the method to extend for a nonlinear decision surface. The new optimization problem is given as

(6) min⁡w,ξ ⁡12||w||2+C∑i=1Nξis.t. yi(w·xi+b)≥1−ξi, ξi≥0,  i=1,2,…,n,

where *C* is a penalty parameter which manages the tradeoff between margin maximization and error minimization. Thus, the classification decision function becomes

(7) $$\begin{array}{c} f\left(x\right)=\mathrm{sign}\left(\sum_{i=1}^{N}L_{i}y_{i}K\left(x_{i},x_{j}\right)+b\right), \end{array}$$

where *L*_*i*_ are Lagrange multipliers, and *K*(*x*_*i*_, *x*_*j*_) = *φ*(*x*_*i*_) · *φ*(*x*_*j*_) is a kernel function which can map the data into a higher dimensional space through some nonlinear mapping function *φ*(*x*) for a nonlinear decision system. In present work, we use radial basis function (RBF) kernel function. Consider two samples *x*_*i*_ = [*x*_*i*1_, *x*_*i*2_,…, *x*_*id*_]^*T*^ and *x*_*j*_ = [*x*_*j*1_, *x*_*j*2_,…, *x*_*jd*_]^*T*^. The RBF kernel is calculated using *K*(*x*_*i*_, *x*_*j*_) = exp⁡(−*γ*||*x*_*i*_−*x*_*j*_||^2^), where *γ* > 0 is the width of Gaussian.

### 2.3. Weighted Support Vector Machines (WSVM)

Traditional SVMs assume that each gene of a sample contributes equally to the tumor classification results. However, this is not desirable since the quality of genes has a significant impact on the performance of a learning algorithm, and the quality of different genes is not the same. In this work, we propose a novel WSVM based on PSO.  Section 2.5 describes this process in more details give the training set {*x*_*i*_, *y*_*i*_}_*i*=1_^*N*^ and the weighted vector *α* ∈ *R* ^*d*^ which fulfills ∑_*i*=1_^*d*^ *α*_*i*_ = 1 for *α*_*i*_ ≥ 0.With respect to (5), this optimization problem can be written as follows:

(8) min⁡ 12||w||2s.t. yi(w.diag⁡(α)·xi+b)≥1, i=1,2,…,n,

where $\mathrm{diag}\left(\alpha \right)=\left[\begin{array}{cccc} \alpha_{1} & 0 & \ldots & 0\\ 0 & \alpha_{2} & \ldots & 0\\ \ldots & \ldots & \ldots & \ldots \\ 0 & 0 & \ldots & \alpha_{d} \end{array}\right]$.

Substituting (8) into (6) yields the following new optimization problem

(9) min⁡w,ξ 12||w||2+C∑i=1Nξis.t. yi(w·diag⁡(α)·xi+b)≥1−ξi,ξi≥0,  i=1,2,…,n∑i=1dαi=1,  αi≥0.

Finally, the classification decision function becomes

(10) $$\begin{array}{c} f\left(x\right)=\mathrm{sign}\left(\sum_{i=1}^{N}L_{i}y_{i}K^{\prime }\left(x_{i},x_{j}\right)+b\right), \end{array}$$

where $K\prime (x_{i},x_{j})=\exp(-\gamma \sqrt{\sum_{k=1}^{d}a_{k}{\left(x_{ik}-x_{jk}\right)}^{2}})$ is the weighted RBF kernel.

### 2.4. Particle Swarm Optimization (PSO)

PSO, proposed by Kennedy and Eberhart [12], is inspired by social behavior among individuals like the birds flocking or the fish grouping. PSO consists of a swarm of particles that search for the best position according to its best solution. During each iteration, every particle moves in the direction of its best personal and global position. The moving process of a particle is described as

(11) vidt+1=w∗vidt+C1·rand·(p  idk−xidt)+C2·rand·(p  gbestt−xidt),Xidt+1=αVidt+xidt,

where *t* denotes the *t*th iteration; *C*_1_ and *C*_2_ are learning factors; rand is positive random number between 0 and 1 under normal distribution. *α* is the constraint factor which can control the velocity weight. *w* denotes the inertial weight coefficient; *x*_*id*_ denotes the velocity of a particle *i*; *v*_*id*_ denotes the velocity of a particle *i*; *p*_*id*_ is the personal best position of particle *i*; *p*_*g*best_ denotes the best one of all personal best positions of all particles within the swarm [19, 20].

### 2.5. Proposed Method

**Figure 1:**
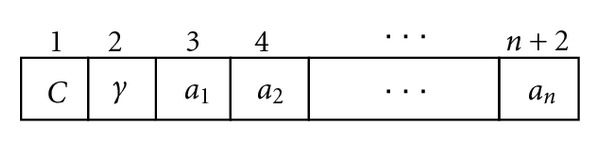
Solution representation.

**Figure 2:**
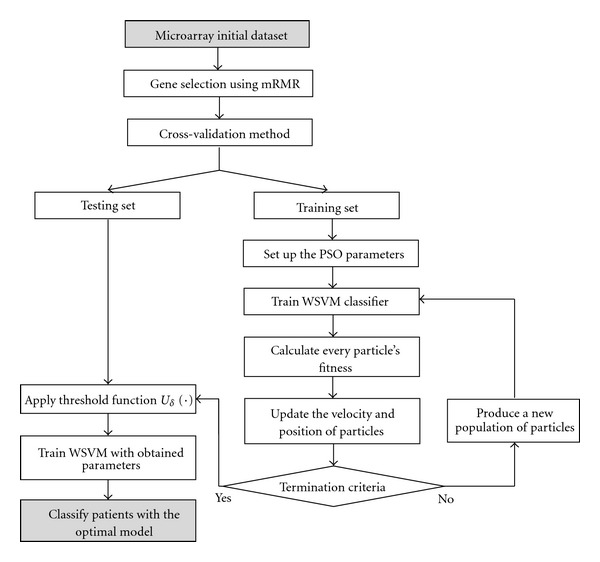
The process of classification by mRMR-PSO-WSVM.

In this section, we introduce the proposed mRMR-PSO-WSVM method. The aim of this system is to optimize the SVM classifier accuracy by automatically (1) preselecting the number of genes using mRMR method, (2) estimating the best gene weights and optimal values for *C* and *γ* by PSO. First, the original microarray dataset is preprocessed by the mRMR filter. Each gene is evaluated and sorted according to mentioned mRMR criterions in Section 3, and the first fifty top-ranked genes are selected to form a new subset. In fact, mRMR is applied to filter out many unimportant genes and reduces the computational load for SVM classifier. Then, a PSO-based approach is developed for determination of kernel parameters and genes weight. Gene weighting is introduced to approximate the optimal degree of influence of individual gene using the training set. Without gene weighting, two decision variables *C* and *γ* are required to be optimized. If *n* genes are required to decide for gene weighting, then *n* + 2 decision variables must be adopted (see Figure 1). The value of *n* variables ranges between 0 and 1, where sum of them is equal to 1. The range of parameter *C* is between 0.01 and 5,000, while the range of *γ* is between 0.0001 and 32.  Figure 2 illustrates the solution representation. We used this representation for particles and allowed PSO to find right value for each variable.

We also define a threshold function *U*_*δ*_(·) to avoid using noisy genes with lower predictive power and to put more importance on the genes with higher discriminative power. In fact,*U*_*δ*_(·) works as gene selector which omits the redundant genes in the final step again. The domain of this function is the set of gene weights and the range is a revised weight for each gene

(12) $$\begin{array}{c} U_{\delta }\left(a_{i}\right)=\{\begin{array}{cc} 0 & \text{if}\;\;a_{i}\leq \delta ,\\ a_{i} & \text{if}\;{\;a}_{i}>\delta , \end{array} \end{array}$$

where 0 ≤ *δ* ≤ 1 and *a*_*i*_ is the degree of importance of *i*th gene. Finally, the weighted vector *α* = (*α*_1_, *α*_2_,…, *α*_*d*_) is determined by normal form as

(13) $$\begin{array}{c} \alpha_{k}=\frac{U_{\delta }\left(a_{k}\right)}{\sum_{i=1}^{d}U_{\delta }\left(a_{i}\right)},\;k=1,2,\ldots ,d. \end{array}$$

Therefore, as mentioned in Figure 2 the training process can be represented as follows

1. Use the mRMR method to preselect fifty top-ranked genes. These selected genes then utilized in next stages where the PSO was employed to obtain optimal gene weights and kernel parameters.
2. Involve the cross-validation method to separate dataset into training and testing set.
3. Then, for each training setset up parameters of PSO. Generate randomly all particles' positions and velocity and set up the learning parameters, the inertia weight and the maximum number of iterations.
4. Train WSVM classifier according to particles values.
5. Calculate the corresponding fitness function formulated by (*classified*/*total*) (*total* denotes the number of training samples, and *classified* denotes the number of correct classified samples) for each particle.
6. Update the velocity and position of each particle using (11).
7. If the specified number of generations is not yet satisfied, produce a new population of particles and return to step (4).
8. Select the gene weights and kernel parameters values from the best global position *p*_*g*best_ and discard redundant genes with threshold function *U*_*δ*_(·).
9. Train WSVM classifier with obtained parameters.
10. Classify patients with the optimal model.

## 3. Experimental Results

The proposed mRMR-PSO-WSVM was implemented using the MATLAB software package version 7.2. We compared our suggested method with SVM, mRMR-SVM, mRMR-PSO-SVM classifiers to consider the effect of each component on classification results. We also extend our experiments by employing the classifiers that have been suggested before by Shen et al, [8] and Abdi and Giveki [18] which were denoted by PSO-SVM^1^ and PSO-SVM^2^ in Table 3, respectively. The discrete PSO was applied to select genes in PSO-SVM^1^. Each particle was encoded to a string of binary bits associated with the number of genes, which is made up of an SVM classifier with all its features. A bit “0” in a particle represented the uselessness of corresponding gene. Also, in PSO-SVM^2^Abdi and Giveki utilized PSO to determine SVM kernel parameters based on the fact that kernel parameters setting in training procedure significantly influence the classification accuracy [18]. 

The classifiers are evaluated on two popular public datasets: leukemia [21] and colon [22] datasets both of which consist of a matrix of gene expression vectors obtained from DNA microarrays for a number of patients. The first set was obtained from cancer patients with two different types of leukemia, acute myeloid leukemia (AML) and acute lymphoblastic leukemia (ALL). The complete dataset contains 25 AML and 47 ALL samples. The second set was obtained from cancerous and normal colon tissues. Among them, 40 samples are from tumors, and 22 samples are from healthy parts of the colons of the same patients [23]. The detailed information of them is collected in Table 1. 

To calculate the accuracy of classifiers, the leave-one-out cross-validation (LOOCV) was involved using a single observation from the original sample as the testing data, and the remaining observations as the training data. This was repeated such that each observation in the sample was used once as the testing data. Moreover, in order to make experiments more realistic, we conducted each experiment 10 times on each dataset, and the average of classification accuracies of ten independent runs besides the average of number of selected genes as considered to evaluate the performance of classifiers. The related parameters of PSO algorithm applied in the experiments are also shown in Table 2.

In addition, we filtered out all those genes having the PSO weight equal to or less than a quality threshold *δ* in the proposed method. To find the best value for this, we started from 0.2 and kept increasing this threshold value by 0.1 and saved the classification results. We found that for leukemia and colon datasets 0.3 ≤ *δ* ≤ 0.5 is always the best choice. Table 3 shows the classification accuracy of classifiers. As it can be observed, the classification accuracy of SVM on two datasets is not very interesting. Furthermore, the accuracy when the mRMR filter is employed generally outperforms the accuracy without gene selection. This implies that gene selection is able to improve the classification accuracy and mRMR is an effective tool to omit the redundant and noisy genes. In addition, the accuracy of PSO-SVM^1^ shows that the selection of genes that are really indicative for tumor classification is a key step in developing a successful gene expression-based data and PSO is a promising tool for handling this. Also, the result of PSO-SVM^2^ emphasizes on the fact that kernel parameters setting significantly influences the classification accuracy of SVM. Classification accuracy of the mRMR-PSO-SVM explains well the benefits of both gene selection and kernel parameters determination using PSO. In final, the proposed mRMR-PSO-WSVM achieves the highest classification accuracy together with lowest average of selected genes on test sets. This confirms that the suggested PSO-based gene weighting achieves better performance compared to binary PSO. Also, the average of selected genes shows that using the threshold function *U*_*δ*_(·) is very effective to reduce the number of selected genes.

Tables 4 and 5 present the results of previously suggested methods besides the proposed mRMR-PSO-WSVM classifier.In order to make a more reliable comparison we try to carry out experiments with two cross-validation methods since some previously reported results were obtained under 10-fold cross validation and the other under LOOCV. Tables 4 and 5 show the results under 10-fold and LOO, respectively.We can see that the proposed classifier can obtain far better classification accuracy than previously suggested methods under both the cross-validation methods. Therefore, we can conclude that our method obtains promising results for gene selection and tumor classification problems.

## 4. Conclusion and Future Researches

This work presented a PSO-based approach to construct an accurate SVM in classification problems dealing with high-deminsional datasets especially gene expressions. This novel approach was a two-stage method in which, first of all, the mRMR filter technique was applied to preselect an effective genesubset from the candidate set. Then it formed a novel SVM in which PSO not only discarded redundant genes, but also especially took into account the degree of importance of each gene and assigned diverse weights to the different genes. It also used PSO to find appropriate kernel parameters since the choice of gene weights influences the optimal kernel parameters and vice versa. The experiments conducted using two different datasets for cancer classification show that the proposed mRMR-PSO-SVM outperforms the previously reported results. Experimental results obtained from UCI datasets or other public datasets and real-world problems can be tested in the future to verify and extend this approach.

## Figures and Tables

**Table 1:** Detailed information of gene expression datasets.

Dataset name		Number of		
		Samples	Categories	Genes
Leukemia	Acute myeloid leukemia	25	2	7129
	Acute lymphoblastic leukemia	47		

Colon	Cancerous colon tissues	40	2	2000
	Normal colon tissues	22		

**Table 2:** PSO parameters.

Parameters	Values
Swarm size	50
The inertia weight	0.9
Accelration constants *C*_1_and *C*_2_	2
Maximum number of iterations	70

**Table 3:** The values of the statistical parameters of the classifiers.

Methods/datasets	Leukemia		Colon	
	Acc (%)	Selected genes	Acc (%)	Selected genes
SVM	90.28	7129	83.87	2000
mRMR-SVM	97.22	50	83.87	50
PSO-SVM^1^	94.44	22.5	85.48	20.1
PSO-SVM^2^	93.06	7129	87.01	2000
mRMR-PSO-SVM	100	17.7	90.32	10.3
*mRMR-PSO-WSVM*	*100*	*3.8*	*93.55*	*6.2*

**Table 4:** Classification accuracy of our method with other methods from literature (under 10-fold cross validation).

(Authors, year)	Method	Leukemia		Colon	
		Acc (%)	S. G.	Acc (%)	S. G.
(Ruiz et al., 2006) [24]	NB-FCBF	95.9	48.5	77.6	14.6
(Shen et al., 2007) [8]	PSOSVM	N. C.	N. C.	91.67	4.00
(Li et al., 2008) [15]	Single PSO	94.6	22.3	87.1	19.8
(Li et al., 2008) [15]	Single GA	94.6	23.1	87.1	17.5
(Li et al., 2008) [17]	Hybrid PSO/GA	97.2	18.7	91.90	18.00
(Shen et al., 2008) [13]	HPSOTS	98.61	7.00	93.32	8.00
*(Abdi et al., 2012) [18] *	*mRMR-PSO-WSVM*	*98.74*	*4.1*	*93.55*	*6.8*

^∗^S. G. and N. C. denote selected genes and not considered, respectively.

**Table 5:** Classification accuracy of our method with other methods from literature (under LOOCV).

(Authors, year)	Method	Leukemia		Colon	
		Acc (%)	S. G.	Acc (%)	S. G.
(Mohamad et al., 2007) [25]	IG + NewGASVM	94.71	20.00	N. C.	N. C.
(El Akadi et al., 2011) [17]	mRMR-GA	100	15.00	85.48	15.00
*(Abdi et al., 2012) [18]*	*mRMR-PSO-WSVM*	*100*	*3.8*	*93.55*	*6.2*

^∗^S. G. and N. C. denote selected genes and not considered, respectively.
